# Autonomic Dysfunction and Blood Pressure Variability in Botulinum Intoxication: A Prospective Observational Study from a Single-Center Italian Outbreak

**DOI:** 10.3390/toxins17040205

**Published:** 2025-04-20

**Authors:** Giuseppe Miceli, Giuliano Cassataro, Vito Volpe, Emanuela Fertitta, Carmelinda Canale, Lucia Tomaiuolo, Melania Blasco, Mariagrazia Stella, Matteo Velardo, Maurizio Renda

**Affiliations:** 1 Department of Health Promotion, Mother and Child Care, Internal Medicine and Medical Specialties (ProMISE), Università degli Studi di Palermo, Piazza delle Cliniche 2, 90127 Palermo, Italy; 2 Internal Medicine and Stroke Care Ward, University Hospital, Policlinico “P. Giaccone”, 90127 Palermo, Italy; 3 U.O.C. of Medicine and Pneumology, Fondazione Istituto G. Giglio, Contrada Pollastra, 90015 Cefalù, Italy; 4 European School of Obstetric Anesthesia, EESOA Simulation Center, 00042 Rome, Italy

**Keywords:** botulin, dysautonomic dysfunction, erectile dysfunction, orthostatic hypotension, toxin

## Abstract

Botulinum neurotoxin (BoNT) intoxication is a rare but severe condition that is characterized by autonomic and neuromuscular dysfunction. This study aimed to evaluate autonomic impairment and blood pressure variability in patients with botulinum intoxication during an outbreak, compared to healthy controls, and to assess their progression over a six-month follow-up period. **Methods:** Twenty (*n* = 20) male patients diagnosed with BoNT intoxication and 34 age- and sex-matched healthy controls were enrolled. At baseline, all subjects underwent 24 h ambulatory blood pressure monitoring (ABPM), and clinostatic and orthostatic blood pressure measurements. Autonomic function parameters, including mean systolic blood pressure (SBP), mean diastolic blood pressure (DBP), SBP and DBP variability, SBP and DBP load, pulse pressure (PP), blood pressure variability ratio (BPVR), and morning surge, were analyzed. Follow-up assessments were conducted after six months. **Results:** Patients with botulinum intoxication exhibited significantly lower SBP, DBP, and blood pressure variability parameters compared to healthy controls. Orthostatic hypotension was present in 55% of patients at baseline, improving to 5% at follow-up. Respiratory failure occurred in 40% of cases, necessitating non-invasive ventilation in 35% and intubation in 20%. At six-month follow-up, mean SBP, DBP, heart rate, and blood pressure variability parameters increased significantly, indicating partial recovery of autonomic control. However, residual abnormalities in autonomic regulation persisted. **Conclusions:** BoNT intoxication leads to notable autonomic dysfunction, marked by impaired blood pressure regulation and a high prevalence of orthostatic hypotension. Although partial recovery occurs, long-term autonomic impairment persists, highlighting the necessity for ongoing cardiovascular monitoring and further research to accelerate autonomic recovery through targeted therapeutic interventions.

## 1. Introduction

Botulin intoxication is a rare neurological syndrome characterized by acute, afebrile, and symmetric descending flaccid paralysis [1]. Italy has one of the highest rates of incidence of botulism in Europe (0.03 cases per 100,000 population), which may be partly attributed to specific aspects of local food culture—such as the widespread tradition of home canning and preserving foods—practices that, when not performed under appropriate hygienic conditions, can occasionally lead to widespread outbreaks [2]. The most common form of the syndrome is represented by foodborne botulism, which is the result of the ingestion of food with preformed toxins produced by *Clostridium botulinum* and other toxin-producing clostridia [1]; four of the seven known different types of botulin neurotoxins (BoNT)—types A, B, E, and F—are toxic for humans [1]. After the gastrointestinal absorption, the BoNTs bind with neuromuscular junctions: the disease typically begins with gastrointestinal disturbances [3] followed by neurological signs, such as impaired motor function and autonomic symptoms [1]. Some patients can experience severe respiratory muscle paralysis, producing pump failure—without gasping or agitation—requiring mechanical ventilation [1,4]. Occasionally, neurological symptoms can be relatively mild, with a predominance of autonomic involvement. Some authors have even reported a pure autonomic failure, with a dry mouth or throat being the earliest signs of botulism [1]. Moreover, cardiovascular autonomic failure in botulism can manifest with marked baroreflex dysfunction, producing higher resting heart rate, supine hypertension, and orthostatic hypotension [5]. Although it has also been suggested that severe autonomic derangement in botulism can be responsible for an augmented risk of cardiac and extra-cardiac complications [6], only a few reports focus on the assessment of cardiovascular reflex functions in botulism. This study was conducted during an outbreak of botulism. Patients were enrolled prospectively throughout the outbreak, and a longitudinal prospective study design was employed. Individuals affected by botulism were compared with a cohort of healthy subjects matched for age and sex. The study aimed to assess the prevalence of hypertension and BoNT-related autonomic disturbances in arterial pressure control among male patients with foodborne botulism. Additionally, it sought to evaluate the 24 h blood pressure monitoring (ABPM) profile both during the acute phase of botulinum toxinfection and six months after toxin ingestion.

## 2. Results

Out of the 30 people suspected to have been affected by the outbreak, 26 were eventually diagnosed with botulism, and, among them, 20 met the inclusion and exclusion criteria. Thirty-four healthy subjects matched for sex and age were enrolled as a control group. The mean age was 48.1 in patients with botulinum and 45.5 in the control group. All patients enrolled in the botulin and control group were male. Table 1 summarizes the frequency of all clinical symptoms in the 20 patients affected by botulinum. Dry mouth and mild dysphagia were the most frequently referred symptoms by patients, respectively, in 80% and 90% of patients. Respiratory failure was present in 8 patients (40%), and almost all of them (*n* = 7) required non-invasive ventilation. Only four patients (20%) underwent intubation and were moved to the Intensive Care Unit. A respiratory failure occurred in our patients on average 7 ± 2 days after ingestion of food contaminated by the toxin. Dysphagia (90%), dysphonia (70%), and dry mouth (80%) were the most frequent symptoms encountered. Erectile dysfunction was a complaint of 65% of patients. Moreover, a strong positive correlation between the number of symptoms and duration of hospitalization was encountered (S = 288.89, *p* < 0.0001), but no specific symptom was associated with prolonged hospitalization (Figure S1).

Demographic and ABPM parameters of botulin patients and healthy subjects are summarized in Table 2. Mean SBP, DBP, and SBP variability, DBP and SBP load, PP, and BPVR were all significantly lower in patients with botulin than the healthy control group. Table 3 summarizes the comparison between ambulatory pressure variables at the time of enrollment and after 6-month follow-up in patients with botulin. Mean SBP, mean DBP, mean HR, and variability of SBP, DBP, and HR 24 h were all significantly increased at the six-month follow-up compared to the values recorded during the hospitalization at t0. SBP and DBP load, PP, and morning surge were significantly augmented as well. During acute intoxication (t0), clinostatic and orthostatic SBP and DBP were significantly lower, with a major rate (55% vs. 5%) of orthostatic hypotension compared to follow-up measurements. Comparison between ABPM parameters in botulin patients at t0 and after follow-up is presented in Figure S2.

## 3. Discussion

The present study comprehensively examines autonomic dysfunction and blood pressure variability in patients affected by botulinum intoxication, comparing their hemodynamic parameters to those of a matched healthy control group and assessing their evolution over a six-month follow-up period. Our findings demonstrate that patients with botulinum intoxication exhibit significantly lower mean SBP, mean DBP, SBP variability, DBP variability, SBP load, DBP load, PP, and BPVR at baseline compared to healthy controls. These results suggest a profound impairment of autonomic regulation of blood pressure during the acute phase of botulinum intoxication. At baseline, a high prevalence of orthostatic hypotension (55%) was observed among affected patients, which substantially improved to 5% at follow-up, indicating partial recovery of autonomic function over time. In botulinum intoxication, orthostatic hypotension is likely due to toxin-induced dysfunction of sympathetic vasoconstrictor pathways, resulting in inadequate compensation for postural changes [7,8]. This finding is consistent with previous reports describing autonomic disturbances in neurotoxin-mediated disorders. The six-month follow-up showed a significant increase in mean systolic and diastolic blood pressure (SBP and DBP), mean heart rate (HR), and the variability of SBP, DBP, and HR, suggesting a progressive restoration of autonomic cardiovascular control. Similarly, increases in SBP and DBP load, PP, and morning surge were also noted. While these parameters did not fully normalize, their improvement indicates a partial autonomic restitution. The persistence of alterations in ABPM parameters highlights the prolonged impact of botulinum toxin on cardiovascular regulation, which warrants further long-term investigations. In a recent article, we demonstrated how, in this group of patients undergoing botulinum intoxication, there was a high incidence of postprandial hypotension, and that this was associated with a seven-fold increased risk of experiencing respiratory failure requiring mechanical ventilation [9]. In this study, however, we highlighted a significant reduction in all parameters of systolic-diastolic blood pressure, orthostatic hypotension, and blood pressure variability in patients affected by botulinum compared to healthy subjects. Furthermore, at the six-month follow-up, we demonstrated an increase in the aforementioned parameters, with normalization of the blood pressure profile and a reduction in orthostaticreduction of orthostatic hypotension. The neurological toxidrome is secondary to the voluntary motor and autonomic cholinergic junction’ blockade. The foodborne form of botulism is a well-known condition in humans, which is characterized by a neurological syndrome resulting from the blockade of voluntary motor and autonomic cholinergic junctions induced by the toxin [10]. This type of botulism occurs when preformed BoNT is ingested through contaminated food. The earliest clinical signs of intoxication may include autonomic symptoms, such as dry mouth or throat, which are often mistaken for pharyngitis [11]. Autonomic disturbance, such as postural hypotension, has also been reported [1]. The clinical features may rapidly worsen in severe cases, leading to respiratory failure (e.g., without gasping or agitation due to muscle paralysis). Mechanical ventilation is often required despite the administration of supportive and specific therapy [12]. Type B botulism may manifest initially as a sixth cranial nerve palsy, while patients with evidence of a third cranial nerve palsy may eventually develop respiratory insufficiency [13].

In our study, the occurrence of a botulinum cluster involving 20 patients has made it possible to reach an unusually large sample considering the epidemiological data on the Italian territory of botulin infection [1]. To the best of our knowledge, this is the largest sample study that has evaluated the autonomic functions of patients with botulinum, with the performance of a clinical and ABPM follow-up.

### 3.1. Clinical Presentation of Autonomic Dysfunction

In our cohort, the most frequent symptoms were dry mouth and mild dysphagia, as already described in the literature [14]. It is interesting to note that a greater number of symptoms at hospital admission was correlated with the length of hospitalization but none of the symptoms found were individually correlated with the length of hospitalization. This is likely related to the dose of toxin taken, which therefore conditions a more severe clinical picture, and, therefore, prolongs the hospital stay. Studies have shown that the amount of toxin ingested correlates with the severity of clinical manifestations, including the need for mechanical ventilation and the duration of hospitalization. For instance, higher doses of botulinum neurotoxin type A result in more prolonged paralysis and a slower recovery process compared to lower doses [14,15].

Additionally, the type of botulinum toxin ingested also plays a role. Botulinum neurotoxin type A generally causes more severe and prolonged symptoms compared to types B and E, which is consistent with clinical observations and laboratory data [16]. Moreover, the presence of comorbidities, especially chronic and inflammatory bowel diseases, could influence the duration and severity of symptoms. Our cohort was very homogeneous for sex and age, and it is composed of young, healthy working persons with no important comorbidities. No cardiovascular risk factors, such as hypertension and diabetes or anamnestic intestinal diseases, were reported in our patients. So, it is plausible that the differences in disease duration and severity could be secondary to the toxin dose absorbed, but, unfortunately, we cannot measure nor estimate the dose assumption with food for each patient. Furthermore, since only male subjects were exposed, this study allowed us to focus attention on the real incidence of some overlooked autonomic symptoms, such as erectile dysfunction, which have only been anecdotally reported in clinical cases [17]. In fact, in our cohort, more than 60% of patients complained of erectile dysfunction upon admission, and many of these reported persistence of such symptoms at the 6-month follow-up. In the past, a syndrome with prevailing autonomic dysfunction derived from foodborne botulism was rarely described [18]. Jenzer et al. [19] reported in 1975 that blurred vision, dry mouth, and constipation were the most common and consistent symptoms. Delayed gastrointestinal transit induced by botulinum toxin may play a crucial role in determining the severity of the intoxication by increasing toxin bioavailability. Sexual dysfunction has only been documented in one case [20]. Botulism may, therefore, be an underestimated cause of pure autonomic dysfunction. BoNT/B likely has a greater affinity for cholinergic autonomic nerve endings than for motor nerve endings [21], resulting in the clinical presentation of predominant autonomic dysfunction.

### 3.2. Blood Pressure Characteristics in Botulin Infection

By performing the ABPM, our study investigated, for the first time, the characteristics of the blood pressure profile and the trend of the main blood pressure variables in patients affected by acute botulism poisoning compared to healthy controls. Although cases of heart rate alterations in patients with botulinum toxin are reported in the literature, it is generally underlined that arterial pressure is not influenced by the toxin activity. Only one study previously reported some fluctuations in pulse and blood pressure of patients with botulinum during an outbreak in Thailand [22]. Our study, on the other hand, demonstrated that patients affected by botulinum present a significant reduction in the mean values of systolic and diastolic blood pressure, as well as a reduced systolic-diastolic pressure load and PP probably as a consequence of a vasodilator mechanism triggered by toxin binding. Interestingly, some recent studies have shown how the administration of botulinum toxin type A can be responsible for vasodilatation through a sympathetic blockade in the skin and stimulate angiogenesis in rats [23]. Moreover, some authors demonstrated that long-term blood pressure control can be achieved by the celiac plexus block with botulinum toxin [24], probably exploiting the decrease in splanchnic norepinephrine and an attenuated hypertensive response to angiotensin II secondary to celiac ganglion block [25]. Unsurprisingly, in our study, patients affected by botulism also presented reduced systolic and diastolic blood pressure variability compared to healthy controls. Several studies demonstrated how blood pressure variability reflects sympathetic activation and impairment of baroceptive reflexes [26]. In this regard, during the acute phase of botulinum toxinfection, our patients showed a higher incidence of orthostatic hypotension than follow-up controls performed in the same patients 6 months later, suggesting once again an autonomic impairment involving the baroreceptor reflexes. There is a possibility that blood pressure variability alteration could be due to the effect of botulinum toxin on vessel walls, but this remains just a speculation. To the best of our knowledge, **no studies have demonstrated alterations in blood pressure variability due to the effect of botulinum toxin on vessel walls.** The studies referenced primarily focus on the vasodilatory effects of botulinum toxin-A (BoNTA) on microcirculation and blood flow in animal models. For instance, Stone et al. demonstrated that BoNTA causes arteriolar vasodilation through the sympathetic blockade, resulting in increased blood flow without affecting systemic arterial pressure [27]. Similarly, Aru et al. found that BoNTA pretreatment induces an increase in resting arteriolar diameter, suggesting a vasodilatory effect [28]. Hayashi et al. reported increased blood flow in the femoral artery following BoNTA administration [29].

However, these studies do not specifically address alterations in blood pressure variability due to BoNTA’s effects on vessel walls. The focus is on localized vasodilation and increased blood flow rather than systemic blood pressure variability. Therefore, while BoNTA has demonstrated effects on microvascular tone and blood flow, there is no evidence in the current medical literature linking BoNTA to changes in blood pressure variability.

## 4. Limitations and Future Perspectives

Our study has several strengths, including its prospective design, standardized measurement protocol, and blinded assessment of blood pressure parameters. However, certain limitations should be acknowledged. The sample size was relatively small, which may limit the generalizability of our findings. Additionally, all patients were male, precluding conclusions regarding potential sex-related differences in autonomic dysfunction associated with botulinum intoxication. The findings of this study open several avenues for future research into the long-term consequences of BoNT intoxication on autonomic function and blood pressure regulation. Given the persistent autonomic impairment observed at six months, further studies should explore the underlying pathophysiological mechanisms driving incomplete autonomic recovery and identify potential biomarkers for long-term dysfunction. Additionally, investigating targeted therapeutic interventions, such as pharmacological treatments or rehabilitative strategies, could provide insights into accelerating autonomic recovery and mitigating residual impairment. Future research should also focus on larger studies to confirm these findings and assess whether individual patient characteristics, such as toxin dose or pre-existing conditions, influence the extent of autonomic dysfunction and recovery. Longitudinal studies with extended follow-up periods are also needed to determine whether autonomic disturbances persist beyond six months and to evaluate their potential impact on long-term cardiovascular health. Finally, future research, particularly studies conducted on animal models, could be valuable in exploring and confirming this association between botulin toxinfection and erectile dysfunction.

## 5. Conclusions

This study demonstrates that botulinum intoxication can induce significant dysfunction in autonomic blood pressure control, characterized by reduced blood pressure variability, impaired orthostatic regulation, and a high prevalence of orthostatic hypotension during the acute phase. Despite partial recovery over six months, residual abnormalities in blood pressure control persist, suggesting prolonged autonomic impairment. These findings emphasize the importance of long-term cardiovascular monitoring in patients recovering from botulinum intoxication to mitigate potential complications associated with autonomic dysfunction.

## 6. Methods

### 6.1. Population

We enrolled patients affected by botulism during an outbreak after ingestion of the same food, altered by botulism neurotoxin (BoNT), admitted to the UOC of Medicine and Pneumology of Istituto Fondazione “G. Giglio” in Cefalù, 90015, Italy. For ethical and privacy reasons—particularly due to the small size of the cohort—we chose not to disclose the specific location, year, and month of the outbreak. This decision was to ensure the confidentiality and anonymity of the patients involved. The main inclusion criterium was the diagnosis of botulism, which was initially performed by clinical suspicion and then confirmed by the isolation of the toxin in the patient’s serum, stool, and food sources. Exclusion criteria were a history of hypertension, type 1 and 2 diabetes mellitus, alcoholism, Parkinson’s disease, and atypical parkinsonism, and all conditions potentially responsible for dysautonomia. All patients underwent the administration of botulin antitoxin within 72 h of diagnosis. The control population consisted of 34 healthy subjects matched by age and sex. The study protocol was conducted in accordance with the Helsinki Declaration, and all patients consented to the study protocol by signing a written informed consent.

### 6.2. Measurement

At the enrollment time, all patients underwent a test for detection of BoNT in stool specimens, ABPM, and blood pressure measurements in clinostatic and orthostatic positions. A trained, blinded physician obtained all blood pressure measurements in the morning. Clinostatic blood pressure was measured in the right arm using an aneroid sphygmomanometer (Heine), with three readings taken at 30 s30-s intervals after the subject had been in a supine position for at least five minutes. The average of these measurements was recorded as the clinostatic blood pressure. Orthostatic blood pressure was subsequently measured immediately upon standing, as well as at 90 and 180 s of orthostasis, by the same operator. They also underwent a daily neurological physic examination and a specific interview about gastroenteric, neurological, and general symptoms linked to BoNT every 4 h. Erectile Dysfunction was evaluated according to Erection Hardness Score [30]. We considered a score from 0 to 2 indicative of erectile dysfunction.

ABPM was performed by using Spacelab SL-90227. The device is provided by an oscillometric record validated and recommended for clinical use. Monitoring equipment was arbitrarily applied at 8:00 am. The cuff was fixed to the non-dominant arm, and three blood pressure readings were taken concomitantly with sphygmomanometer measurements to ensure that the average of the two sets of values did not differ by >5 mmHg. The device was set to measure blood pressure at 15 min15-min intervals during the day (06:00 am—10:00 pm) and at 30 min30-min intervals during the night (10:00 pm to 06:00 am). During the 24 h of examination, patients were told to keep their arms immobile at the time of the measurements. The patient had no access to the ambulatory blood pressure values. During the blood pressure monitoring patients were asked to carry out, as far as possible during hospitalization, an active lifestyle avoiding physical effort and consumption of caffeine-based beverages. Patients were also asked to note main meal times, fall asleep, and wake-up times during the 24 h-monitoring. The following parameters were calculated by ABPM Spacelab SL-90227 software analysis: mean systolic blood pressure (SBP), mean diastolic blood pressure (DBP), mean heart rate (HR), SBP Variability, DBP Variability, SBP load, DBP load, pulse pressure (PP), Ambulatory Arterial Stiffness Index (AASI), blood pressure variability ratio (BPVR), morning surge. Blood Pressure load was defined as the percentage of systolic/diastolic readings ≥135/85 and ≥120/70 mm Hg during the day and night, respectively, respectively [31].

Morning surge was calculated as the blood pressure 2 h after rising minus the average blood pressure during sleep.

ABPM examination and clinostatic and orthostatic blood pressure measurements were performed sequentially in random order, and 60% of the patients underwent ABPM first. All examinations were performed before the occurrence of severe clinical manifestations like respiratory failure and severe dysphagia. Autonomic tests were executed in a room with a controlled temperature (25 °C) and minimal external noise. Hypertension was defined in accordance with ESC guidelines [32]. In agreement with the guidelines of the Consensus Committee of the American Autonomic Society and American Academy of Neurology, orthostatic hypotension was defined as a drop in blood pressure of at least 20 mmHg for systolic blood pressure or at least 10 mmHg for diastolic blood pressure within 3 min of standing up [33,34].

After six months, all patients underwent a clinical and instrumental follow-up, including ABPM, measurements of clinostat and orthostatic blood pressure according to the aforementioned protocol, and ECG. Patients were also reinterviewed about the evolution of their signs and symptoms.

### 6.3. Statistical Analysis

Statistical analysis was conducted for both quantitative and qualitative data, incorporating descriptive statistics for all variables. A post hocpost-hoc power analysis was performed to ensure that the sample size (study group: 20; control group: 34) provided a significance level of 95% and a test power of 80% for the applied models. The Shapiro–WilkShapiro-Wilk test was used to assess the normality of the studied variables. Comparisons between the two groups and within the study group after follow-up were conducted using unpaired *t*-tests for parametric data and Wilcoxon tests for non-parametric data. A Spearman correlation analysis was conducted to assess the relationship between the number of symptoms and the duration of hospitalization. Simple and multinomial logistic regression models were applied to explore potential dependencies among the variables in the control and study groups and their association with observation time. Additionally, a mixed-effects repeated measures ANOVA model was employed to evaluate changes in the studied metrics over time and between groups. Logistic regression models were further used to assess potential relationships between risk factors and explanatory variables. Continuous data are presented as mean ± standard deviation (SD) unless otherwise specified. All *p*-values were two-tailed, with statistical significance set at *p* ≤ 0.05.

## Figures and Tables

**Table 1:** List of patients affected by the outbreak-associated botulism and the frequency of each clinical variable.

Patients	Age	Hosp. (days) days(days)	N Symtom	B.V.	Diplopia	Ptosis	Mydriasis	Dry mouth	Dysphagia	Dysphonia	Dysarthria	Constipation	Diarrhea	Nausea	Ataxia	Asthenia	M.H.	U.R.	R.F	N.F.V.	Intubation	E.D.
#1	45	22	9	-	-	+	-	+	+	+	+	-	-	+	-	+	+	-	+	+	+	+
#2	53	25	9	+	+	+	-	+	+	+	-	+	-	+	-	-	+	-	+	+	+	-
#3	48	18	6	+	-	+	-	+	+	-	-	-	-	-	-	+	-	-	-	-	-	+
#4	51	14	4	-	-	-	-	+	+	+	-	-	-	-	-	+	-	-	-	-	-	-
#5	41	8	6	+	+	-	-	+	+	+	-	-	-	-	-	-	-	-	-	-	-	+
#6	32	9	3	-	-	-	-	+	+	-	-	-	-	-	-	-	-	-	-	-	-	+
#7	57	18	7	+	-	-	+	+	+	-	-	+	-	-	-	+	-	-	-	-	-	+
#8	54	20	7	+	-	-	-	+	+	+	-	-	-	-	-	+	+	-	-	-	-	+
#9	52	14	3	-	-	-	-	-	+	+	-	-	-	-	-	-	-	-	-	-	-	-
#10	42	7	2	-	-	-	-	-	-	-	-	-	-	-	-	-	+	-	-	-	-	+
#11	51	9	4	-	-	-	-	+	+	+	-	-	-	-	-	+	-	-	-	-	-	+
#12	49	24	11	+	+	+	+	+	+	+	+	-	-	-	+	+	-	-	+	+	+	+
#13	28	12	5	-	-	-	-	+	+	+	-	-	+	-	-	+	-	-	+	+	-	-
#14	48	7	2	-	-	-	-	-	+	+	-	-	-	-	-	-	-	-	-	-	-	-
#15	59	17	5	+	+	-	-	-	+	+	+	-	-	-	-	-	-	-	+	+	-	-
#16	42	22	11	+	+	+	+	+	+	+	-	-	-	-	+	+	+	-	+	+	-	+
#17	52	19	3	-	-	-	-	+	-	+	-	-	-	-	-	-	-	+	+	-	-	+
#18	39	12	5	-	+	-	-	+	+	-	-	+	-	-	-	-	-	-	-	-	-	+
#19	59	7	3	-	-	-	-	+	+	-	-	-	-	-	-	-	-	-	-	-	-	+
#20	60	20	5	-	-	-	-	+	+	+	-	+	-	-	-	-	+	-	+	+	+	-
%				40	30	25	15	80	90	70	15	20	5	10	10	45	30	5	40	35	20	65

B.V.: blurred vision; E.D.: erectile dysfunction; M.H.: Muscular Hyposthenia; N.F.V.: need for ventilation; R.F.: respiratory failure; U.R.: Urinary Retention. +: presence of the symptom, -: absence of the symptom.

**Table 2:** Demographic and ambulatory pressure variables in patients affected by botulinum and in healthy control group.

Variables	Bot. Group (*N* = 20)	Control Group (*N* = 34)	*p* Value
Age	48.1 ± 8.72	45.5 ± 11.22	0.347
Sex M; F (%)	100; 0	100; 0	
Mean SBP	109.35 ± 7.83	116.32 ± 8.08	**0.003**
Mean DBP	71.5 ± 6.81	74.24 ± 7.1	0.168
Mean HR	70.5 ± 8.68	69.18 ± 7.12	0.568
SBP Variability	10.76 ± 1.82	12.77 ± 2.9	**0.003**
DBP Variability	8.83 ± 1.78	10.29 ± 2.49	**0.015**
HR 24 h Variability	9.15 ± 2.35	9.44 ± 2.85	0.688
SBP Load	2 (1; 5)	10 (2; 20)	**0.006**
DBP Load	11 (6; 25)	26 (13; 45)	**0.052**
Pulse Pressure	37.65 ± 4.78	42.68 ± 6.91	**0.003**
AASI	0.34 ± 0.09	0.34 ± 0.14	**0.001**
BPVR	1.23 ± 0.11	1.39 ± 0.23	0.631
Morning Surge	11.12 ± 9.2	9.94 ± 7.68	
Hypertension (%)	0	0	

The table shows the mean (±standard deviation) or median (Q1; Q3) summary indexes of the quantitative variables under study. AASI: Ambulatory Arterial Stiffness Index; BPVR: blood pressure variability ratio; DBP: diastolic blood pressure; HR: heart rate; SBP: systolic blood pressure.

**Table 3:** Ambulatory pressure variables at the time of enrollment and after a follow-up of 6 months in patients with botulin intoxication.

Variables	t = 0	t = 6	*p* Value
Mean SBP	109.35 ± 7.83	119.05 ± 11.23	**0.0001**
Mean DBP	71.5 ± 6.81	76.75 ± 7.9	**0.001**
Mean HR	70.5 ± 8.68	74.55 ± 10.37	**0.010**
SBP Variability	10.76 ± 1.82	11.97 ± 3.39	**0.050**
DBP Variability	8.83 ± 1.78	9.94 ± 2.81	**0.030**
HR 24 h Variability	9.15 ± 2.35	10.91 ± 4.23	**0.018**
SBP Load	4.81 ± 6.82	19.52 ± 22.6	**0.008**
DBP Load	18.12 ± 20.7	33.54 ± 29.36	**0.020**
Pulse Pressure	37.65 ± 4.78	42 ± 6.07	**0.0001**
AASI	0.34 ± 0.09	0.33 ± 0.08	0.296
BPVR	1.23 ± 0.11	1.25 ± 0.18.	0.0553
Morning Surge	11.12 ± 9.2	27.04 ± 21.52	**0.004**
Clinostatic SBP	115.26 ± 12.64	128.21 ± 16.01	**0.007**
Clinonostatic DBP	67.89 ± 7.69	76.84 ± 10.05	**0.003**
Ortostatic SBP 1 min	101.84 ± 14.55	126.16 ± 20.47	**0.0001**
Ortostatic DBP 1 min	73.68 ± 11.77	80.84 ± 14.38	**0.015**
Ortostatic SBP 1.5 min	106.05 ± 14.77	123.89 ± 19.85	**0.001**
Ortostatic DBP 1.5 min	72.11 ± 15.57	78.47 ± 16.27	**0.035**
Ortostatic SBP 3 min	107.63 ± 18.44	125.68 ± 23.39	**0.001**
Ortostatic DBP 3 min	72.37 ± 15.76	79.74 ± 16.8	**0.031**
Ortostatic Hypotension (%)	55%	5%	**0.0001**

AASI: Ambulatory Arterial Stiffness Index; BPVR: blood pressure variability ratio; DBP: diastolic blood pressure; HR: heart rate; SBP: systolic blood pressure.

## Data Availability

The raw data supporting the conclusions of this article will be made available by the authors on request.

## Supplementary Materials

The following supporting information can be downloaded at: https://www.mdpi.com/article/10.3390/toxins17040205/s1, Figure S1: Relation between number of symptoms and hospitalization; Figure S2: Comparison between ABPM parameters in botulin patients and control group at t0 and after follow-up.

## Abbreviations

The following abbreviations are used in this manuscript:

AASI	Ambulatory Aarterial Sstiffness Iindex
ABPM	Ambulatory Blood Pressure Monitoring
BoNt	Botulin Neurotoxin
HR	Heart Rate
PP	Pulse Pressure
SBP	Systolic Blood Pressure
