# Supplementary figures and images for: Autonomic Dysfunction and Blood Pressure Variability in Botulinum Intoxication: A Prospective Observational Study from a Single-Center Italian Outbreak
Source: Toxins (Basel). 2025 Apr 20;17(4):205. doi: 10.3390/toxins17040205 (PMC12031082; doi:10.3390/toxins17040205)

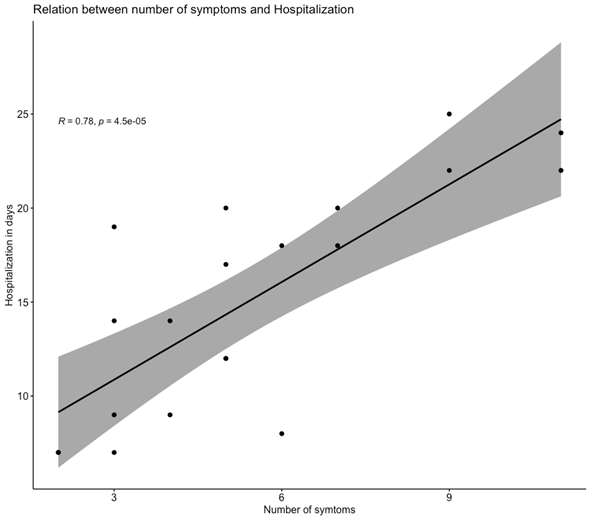

Supplement: Supplementary file 1 [file toxins-17-00205-s001.zip › Figure S1.png]
